# NEAT HFpEF: Organic nitrates fail to deliver

**DOI:** 10.21542/gcsp.2016.1

**Published:** 2016-03-31

**Authors:** Kerolos Wagdy, Mohamed Hassan

**Affiliations:** Aswan Heart Centre, Aswan, Egypt

## Introduction

Heart failure with preserved ejection fraction (HFpEF) has emerged as a major cause of cardiovascular morbidity and mortality in last decades.^1^ The incidence of HFpEF continues to rise and currently it accounts for approximately half of all patients with HF, with morbidity, mortality, and health care costs on par with HF with reduced EF (HFrEF).^1,2^ HFpEF remains among the most challenging of clinical syndromes for the clinician and scientist, with a multitude of proposed mechanisms involving the heart and other organs and complex interplay with common co-morbidities.^2^ Unfortunately and to date, there is no effective form of therapy for such patients. The therapeutic picture for HFpEF has been less favorable than HFrEF. Other than an emphasis on rigorous control of hypertension, atrial fibrillation, and fluid retention, there is relatively little new that can be offered to those patients. Regular exercise is one of the few interventions which may offer modest improvement in peak exercise capacity in such patients – as evident in the PARIS study.^3^ The apparent failure of pharmacotherapy stems from the fact that HFpEF can be multifactorial and the molecular pathways remain largely undefined.^1^

## Nitrates and HF

**Figure 1.**
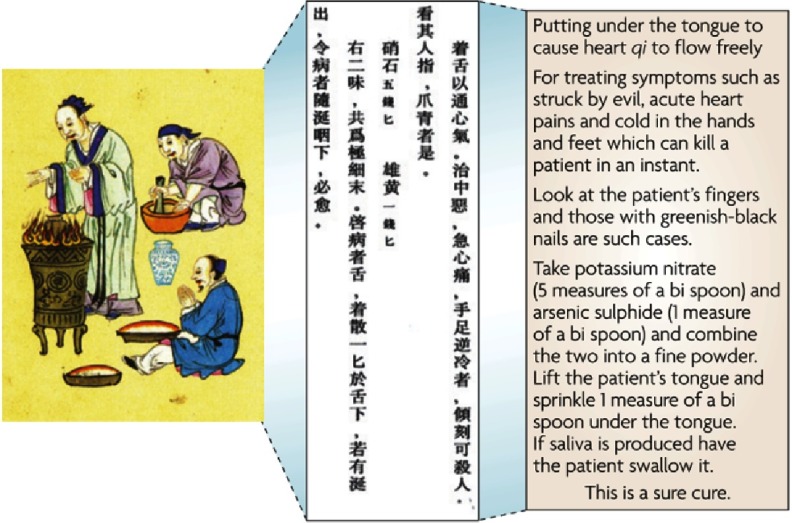
A medical recipe from Dunhuang (western China). The therapeutic benefits of nitrate as evidenced by a translation of medieval Buddhist manuscripts. It illustrates the early appreciation of the effect of nitrate, readily available for meat curing and gunpowder and reduced to nitrite in saliva, on cardiovascular conditions (angina and digital ischaemia). The term qi refers to a ‘fluid’ that, in a healthy person, flows harmoniously around the body. Its flow is disrupted during sickness. A bi spoon was a ceremonial spoon used in medicine. Chinese physicians often added realgar (arsenic sulphide) to a recipe as its colour is that of healthy blood. It would have had no effect because of its low solubility (adapted from reference [8]).

The therapeutic benefits of nitrates in cardiovascular medicine have been known for a long time, even during the medieval period (Figure 1). According to the most recent American College of Cardiology (ACC) / American Heart Association (AHA) 2013 guidelines, the combination of isosorbide dinitrate and hydralazine is recommended to reduce morbidity and mortality for African Americans with HFrEF who remain symptomatic despite optimal therapy with ACE inhibitors and beta blockers (class I recommendation)^4^ and may be considered as a therapeutic option for those who are intolerant of ACE inhibitors or ARB (class IIa recommendation).^5^ Despite lack of clinical evidence, nitrates are also commonly prescribed by practicing clinicians to improve exercise capacity in patients with HFpEF. Approximately 15 to 50% of patients with HFpEF are treated with nitrates,^6,7^ however it may predispose them to excessive hypotension and other adverse effects.

## Nitrate, nitrite, and nitric oxide cycle

Nitric oxide (NO) is a critical regulator of vascular homeostasis, neurotransmission, and home defense.^9^ It is mainly synthesized from the amino acid L-arginine by a family of NO synthase (NOS) enzymes. The synthesis of NO by vascular endothelium via endothelial NOS (eNOS) is responsible for the vasodilator tone that is essential for regulation of vascular homeostasis and blood pressure.^9,10^ In the central nervous system, NO – synthesized by neuronal NOS (nNOS) - is a neurotransmitter involved in several brain functions including the formation of memory. Moreover a network of non-cholinergic non-adrenergic nerves operate through NO dependent mechanism which mediate some of the neurogenic vasodilation and regulate various gastrointestinal, respiratory, and genitourinary functions. ^9^ In systemic inflammatory disorders such as sepsis and severe gastroenteritis, NO level is greatly increased owing to up regulation of inducible NOS (iNOS) activity.^11^

Nitrite (NO_2_^−^) and nitrate (NO_3_^−^) are the oxidative end products of endogenous nitric oxide (NO) metabolism. In addition, both can be provided for in diet. Cured meat and bacon are rich in NO_2_^−^, while many vegetables are rich in NO_3_^−^. Interestingly, a plate of green leafy vegetables such as lettuce or spinach contains more NO_3_^−^ than is formed *in vivo* by NOS.^8^

**Figure 2.**
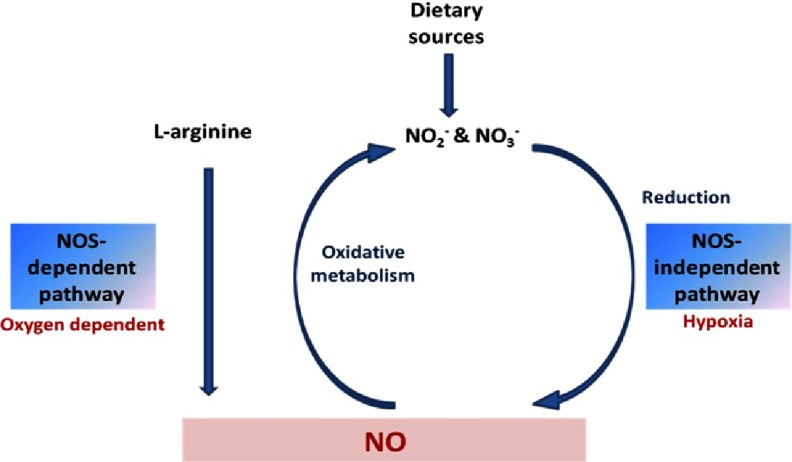
Nitric oxide, nitrite, and nitrate cycle.

Despite being recognized for a long time as inert products, NO_2_^−^ and NO_3_^−^ represent a stable reservoir for NO. Both can be physiologically recycled and reduced to form bioactive NO in blood and tissues, see the “NOS-independent pathway” (Figure 2).^8,12^

**Figure 3.**
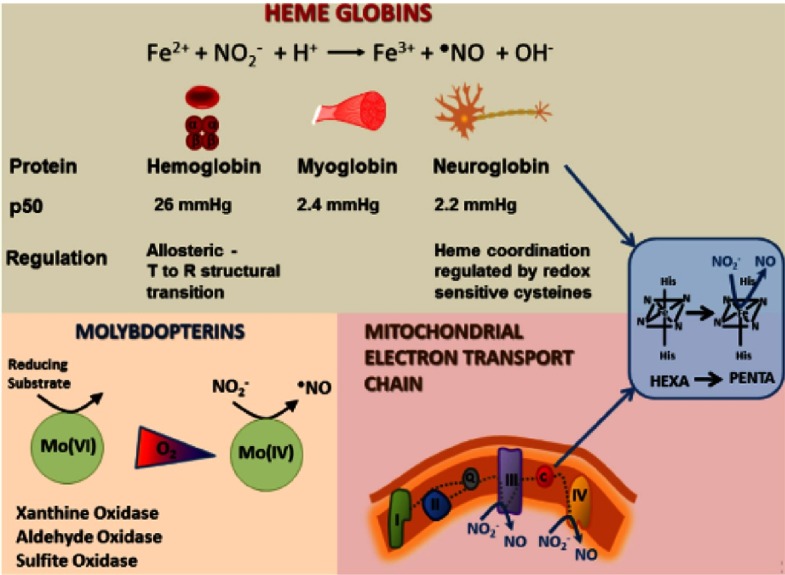
Major classes of nitrite reductase (adapted from reference [12]).

Due to lack of specific and effective NO_3_^−^ reductase enzymes in human, NO_3_^−^ should be initially reduced to NO_2_^−^ by the commensal bacteria in the oral cavity and gastrointestinal tract, and to a lesser extent by xanthine oxidoreductase in the liver.^13–15^ Then numerous pathways involving haemoglobin (in blood),^16^ myoglobin (in muscles),^17^ neuroglobin (in brain and retina),^18^ molybdenum-containing enzymes (xanthine oxireductase, aldehyde oxidase, and sulfite oxidase),^19^ and components of the mitochondrial electron transport chain^20,21^ are involved for further reduction to NO (Figure 3).

All these pathways require low oxygen tensions to effectively generate NO. Therefore the generation of NO by this pathway is greatly enhanced during hypoxia and acidosis, ensuring NO production in situations for which the oxygen-dependent NOS enzyme activities are compromised.^8,22^ This appears to contribute to the physiological hypoxic vasodilation, and has been implicated in protection against myocardial damage after ischemia/reperfusion (I/R) in the heart, which is largely mediated by inhibition of mitochondrial complex I and IV.^17,23^

The biological activities of the inorganic NO_2_^−^ and NO_3_^−^ must be distinguished from the anti-anginal organic nitrates (nitroglycerin) and nitrites (amyl-nitrite). Organic nitrates and nitrites are much more potent than NO_2_^−^ in terms of vasoactivity. The *in-vivo* bioactivation of organic nitrates requires metabolism into NO_2_^−^ and NO by mitochondrial aldehyde dehydrogenase and other enzymes.^24^

The overall effect of nitrates in patients with HFpEF is uncertain and not extensively studied. The Nitrate’s Effect on Activity, Tolerance in Heart Failure with Preserved Ejection Fraction (NEAT-HFpEF) trial (presented at the AHA 2015 scientific sessions and simultaneously published in the *New England Journal of Medicine Journal* in November 2015) provides insights into the overall effect of nitrates on activity tolerance in such patients.^25^

## NEAT-HFpEF

The NEAT-HFpEF study is a multicenter, double blinded, placebo control and crossover trial which was conducted to investigate the role of isosorbide mononitrates in the management of HFpEF.^25^

**Figure 4.**
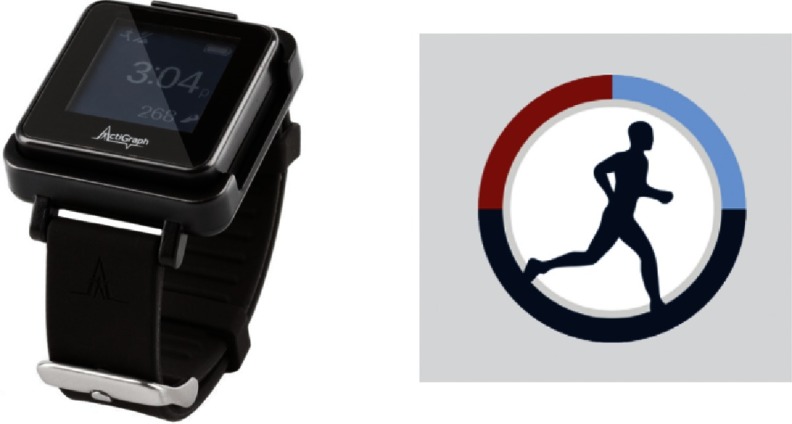
ActiGraph GT9X Link Accelerometer.

The study enrolled 110 ambulatory patients with HFPEF (ejection fraction of 50% or more), 50 years old or more, who were randomly divided into two groups with subsequent crossover. The first group received gradually uptitrated isosorbide mononitrates from 30 mg to 60 mg to 120mg once daily for 6 weeks, while the other group received placebo for 6 weeks with subsequent crossover to each other group for 6 weeks. Each group was assessed for the daily activity level with accelerometer units during the 120mg phase as a primary end point (Figure 4). The secondary end points included hours of activity during the 120-mg phase, daily accelerometer units during all three dose regimens, quality of life, 6-minute walk distance, and levels of N-terminal pro brain natruretic peptide (NT-proBNP).

**Figure 5.**
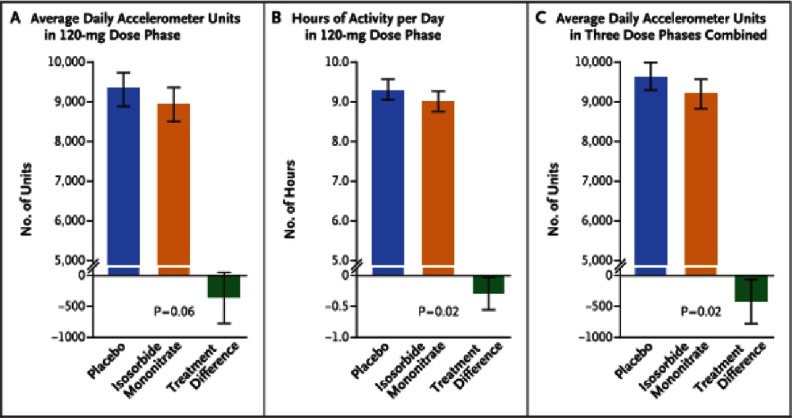
Primary and secondary end points for activity levels.

In the group receiving the 120-mg dose of isosorbide mononitrate, as compared with the placebo group, there was a non-significant trend toward lower daily activity [−381 accelerometer units; 95% confidence interval (CI): −780 to 17; P = 0.06] and a significant decrease in hours of activity per day (−0.30 hours; 95% CI, −0.55 to −0.05; P = 0.02) (Figure 5). Activity levels decreased progressively and significantly with increased doses of isosorbide mononitrate (but not placebo). There were no significant differences in the 6-minute walk distance, quality-of-life scores, or NT-proBNP levels between the two groups. Numerically, more patients discontinued isosorbide mononitrate than placebo.

## Discussion

Isosorbide mononitrate did not improve the daily activity level, exercise capacity, quality of life, or NT-proBNP levels in patients with HFpEF. In contrast to its effects in patients with HFrEF, nitrates were associated with a dose-dependent decline in daily activity levels in patients with HFpEF. Patients who received nitrates had numerically lower activity time, quality of life scores and higher NT-proBNP. They also showed statistically significant reductions in systolic blood pressure, compared to placebo group. This may be related to the pathophysiologic differences between the two HF disorders. Increased ventricular and vascular stiffness, autonomic dysfunction, chronotropic incompetence, and altered baroreflex sensitivity – common in HFpEF – may limit the hemodynamic benefits of nitrates.^26^

The current study provides a very strong signal that nitrates have no benefit and may be detrimental in patients with HFpEF. However, NEAT-HFpEF is not a very large study. It included 110 patients only, and there is still no consensus regarding the entry criteria for patients with HFpEF in all trials.^27^ Furthermore, the results may have been limited by the rapid dose escalation of nitrate therapy. This class of patients are relatively sensitive to changes in hemodynamics, thus more gradual dose escalation might have yielded different results.

The assessment of daily activities using patient-worn accelerometers is a unique feature in the current study. It is an excellent objective measure of exercise tolerance and it provides unique quantitative information about the impact of therapies on patients’ daily functional status, rather than intermittent repetition of coached exercise tests or memory-dependent quality-of-life questionnaires.^28^

The decline in daily activity levels (18 minutes per day) which was observed in the nitrates group is clinically meaningful. Prior observational studies have shown that even a 10-minute reduction in activity per day in patients with HFrEF was associated with adverse outcomes.^29^

## What have we learned?

Empiric use of long-acting nitrates to improve symptoms in HFpEF is not beneficial and it may have adverse effects on exercise tolerance. Inorganic nitrite or nitrate may enhance nitric oxide bioavailability preferentially during exercise and may be more effective and have fewer side effects than organic nitrates.
